# Phylogenomics and systematics in *Pseudomonas*

**DOI:** 10.3389/fmicb.2015.00214

**Published:** 2015-03-18

**Authors:** Margarita Gomila, Arantxa Peña, Magdalena Mulet, Jorge Lalucat, Elena García-Valdés

**Affiliations:** ^1^Microbiology, Department of Biology, Universitat de les Illes BalearsPalma de Mallorca, Spain; ^2^Institut Mediterrani d'Estudis Avançats (Consejo Superior de Investigaciones Científicas-Universidad de las Islas Baleares)Palma de Mallorca, Spain

**Keywords:** *Pseudomonas*, genomics, multilocus sequence analysis, taxonomy, systematics

## Abstract

The genus *Pseudomonas* currently contains 144 species, making it the genus of Gram-negative bacteria that contains the largest number of species. Currently, multilocus sequence analysis (MLSA) is the preferred method for establishing the phylogeny between species and genera. Four partial gene sequences of housekeeping genes (16S rRNA, *gyrB*, *rpoB*, and *rpoD*) were obtained from 112 complete or draft genomes of strains related to the genus *Pseudomonas* that were available in databases. These genes were analyzed together with the corresponding sequences of 133 *Pseudomonas* type strains of validly published species to assess their correct phylogenetic assignations. We confirmed that 30% of the sequenced genomes of non-type strains were not correctly assigned at the species level in the accepted taxonomy of the genus and that 20% of the strains were not identified at the species level. Most of these strains had been isolated and classified several years ago, and their taxonomic status has not been updated by modern techniques. MLSA was also compared with indices based on the analysis of whole-genome sequences that have been proposed for species delineation, such as tetranucleotide usage patterns (TETRA), average nucleotide identity (ANIm, based on MUMmer and ANIb, based on BLAST) and genome-to-genome distance (GGDC). TETRA was useful for discriminating *Pseudomonas* from other genera, whereas ANIb and GGDC clearly separated strains of different species. ANIb showed the strongest correlation with MLSA. The correct species classification is a prerequisite for most diversity and evolutionary studies. This work highlights the necessity for complete genomic sequences of type strains to build a phylogenomic taxonomy and that all new genome sequences submitted to databases should be correctly assigned to species to avoid taxonomic inconsistencies.

## Introduction

The genus *Pseudomonas* is one of the most complex bacterial genera and is currently the genus of Gram-negative bacteria with the largest number of species; in fact, the number of species in the genus has increased every year (10 additional species in 2013 and six in 2014 through October). The current number of recognized and validly published species is 144, including 10 subspecies; these species are present in the List of Prokaryotic Names with Standing in Nomenclature (Parte, 2014).

The taxonomy of the genus has evolved simultaneously with the available methodologies since its first description. The genus *Pseudomonas* was described by Migula in 1894 according to the morphological characteristics of its members (Migula, 1900). For many years, the genus comprised many species that were not always well-characterized until the work of Stanier et al. (1966) in which the physiological and biochemical properties clearly established the taxonomical basis for the identification of the species. In 1984, the genus was revised, and a subdivision of five groups was implemented based on the DNA–DNA hybridisation (DDH) and rRNA-DNA hybridisation results (Palleroni, 1984). Later, the five groups were recognized as being associated with the class *Proteobacteria* (De Vos and De Ley, 1983; De Vos et al., 1985, 1989; De Ley, 1992); the members of the genus *Pseudomonas* “sensu stricto” were shown to belong to rRNA-DNA group I in the subclass *Gammaproteobacteria*. Since then, several authors have reviewed the taxonomic status of the genus *Pseudomonas* (Moore et al., 1996; Anzai et al., 2000; Peix et al., 2009; Mulet et al., 2012a). The approved list of bacterial names (Skerman et al., 1980) included 96 *Pseudomonas* species; however, only 31 of those species are considered true species in the genus *Pseudomonas* in the accepted taxonomy.

Although the 16S rRNA gene is the basic tool of the current bacterial classification system, it is known that closely related species of bacteria cannot be differentiated based on this gene. Therefore, over the past 10 years, other gene sequences have been used as phylogenetic molecular markers in taxonomic studies, such as *atpD*, *gyrB*, *rpoB*, *recA*, and *rpoD* (Yamamoto and Harayama, 1998; Hilario et al., 2004; Tayeb et al., 2005, 2008). Mulet and collaborators have demonstrated that the analysis of the sequences of four housekeeping genes (16S rRNA, *gyrB*, *rpoB*, and *rpoD*) in all known species of the genus clarified the phylogeny and greatly facilitated the identification of new strains (Mulet et al., 2012a; Sánchez et al., 2014). The multilocus sequence analysis (MLSA) approach based on the sequence analysis of the four housekeeping genes has proven reliable for species delineation and strain identification in *Pseudomonas* (Mulet et al., 2012b).

Whole-genome sequences can provide valuable information on the evolutionary and taxonomic relationships in bacteriology. In 2005, Coenye et al. (2005) published an article entitled “Toward a prokaryotic genomic taxonomy” and presented an overview of available approaches to assess the taxonomic relationships between prokaryotic species based on complete genome sequences. These genomic methods are delineated to substitute the experimental DDH by providing the possibility of creating accumulative databases of whole genome sequences. The digital methods used in the genome comparisons for the species delineation in several bacterial genera have been recently discussed by Li et al. (2015) and Colston et al. (2014). The methods tested in the present study, applied to strains in the genus *Pseudomonas*, were: tetranucleotide usage patterns (TETRA; Teeling et al., 2004), average nucleotide identity (ANIm and ANIb; Goris et al., 2007), and genome-to-genome distance (GGDC; Meier-Kolthoff et al., 2013).

Genome sequencing is expected to provide a relevant tool in bacterial taxonomy, and results obtained in the analysis of *Pseudomonas* species will assist in validating the proposed methods of comparison. Next-generation sequencing (NGS) currently provides an exponentially increasing number of whole genome sequences of bacterial strains, and the sequences are used frequently for comparative analyses from which phylogenetic or evolutionary conclusions are drawn. The correct strain assignation to a known species, however, is essential for correct conclusions. Many of the *Pseudomonas* strains under study were isolated and classified several years ago, and their correct taxonomic position is frequently dubious with the current taxonomic tools. The need for a taxonomical revision of several strains defined as different species of the genus *Pseudomonas* was evident when the strains were analyzed with a MLSA using the combined genes *atpD*, *carA*, *recA*, and 16S rRNA (Hilario et al., 2004) or using *gyrB*, *rpoD*, and 16S rRNA (Yamamoto et al., 2000; Mulet et al., 2010). In fact, Mulet et al. (2013) performed a taxonomical revision of *P. putida* strains based on a MLSA with the combined genes 16S rRNA, *gyrB*, and *rpoD*. Their results demonstrated that strains assigned to biovar A of the species were located in the *P. putida* group although not all belonged to the species *P. putida*. Biovar B strains were scattered among six subgroups of the *P. fluorescens* group and also belonged within the *P. putida* group.

Four partial gene sequences of housekeeping genes (16S rRNA, *gyrB*, *rpoB*, and *rpoD*) were obtained from 112 complete or draft genomes of strains related to the genus *Pseudomonas* that were available in databases until December 2012. These genes were analyzed together with the corresponding sequences of 133 *Pseudomonas* type strains of validly published species to assess their correct phylogenetic assignations. Because of the complex taxonomical relationships among species and pathovars in the *P. syringae* phylogenetic group, only six of the 63 available complete *P. syringae* genomes were considered. The *P. syringae* species complex has been studied at the intraspecies level in a recent publication with similar methodology (Marcelletti and Scortichini, 2014).

The main objectives of the present study were: (i) to infer the phylogeny and taxonomic affiliation of the 112 whole genome sequenced strains in the existing taxonomy of the genus *Pseudomonas*; (ii) to compare MLSA with the genome-based methods for species delineation (TETRA, ANIb, ANIm, and GGDC); and (iii) to compare the genome-based methods against each other.

## Materials and methods

### Data collection and genome selection

A total of 253 *Pseudomonas* strains were analyzed in this study, comprising 112 complete or draft genomes of the *Pseudomonas* strains available in the databases and 141 strains of validly published *Pseudomonas* species (Mulet et al., 2012a). Those 141 taxonomically well-characterized strains included 133 *Pseudomonas* type strains, two subspecies of *Pseudomonas chlororaphis* (*P. chlororaphis* subsp. *aurantiaca* and *P. chlororaphis* subsp. *aureofaciens*) and *Pseudomonas pseudoalcaligenes*, the later synonym of *Pseudomonas oleovorans* subsp. *oleovorans* (Saha et al., 2010). In addition to the type strains, four taxonomically well-characterized strains of the *Pseudomonas stutzeri* phylogenetic group were also included: two strains of the species *P. stutzeri* (both members of the genomovar 1, ATCC 27951 and A15) and two strains of *Pseudomonas balearica* (LS401 and st101). “*Pseudomonas alkylphenolia*” JCM 16553 was also included although it has no standing in the nomenclature (Veeranagouda et al., 2011). The set of 112 genome sequences of *Pseudomonas* was retrieved from the Genbank database on 31st December of 2012. All complete and draft genomes not taxonomically identified as members of the *P. syringae* group were included in the analysis. Six genomes affiliated with the *P. syringae* group were also selected. Genomes that did not contain the full-length 16S rRNA, *gyrB*, *rpoB*, and *rpoD* genes sequences were removed from the dataset. The list of the 112 complete or draft genomes analyzed is shown in Supplementary Table 1.

### Multilocus sequence analysis

The sequences of the 16S rRNA, *gyrB*, *rpoB*, and *rpoD* genes were extracted from each complete genome studied and were compared with the corresponding sequences of all species type strains described until 2012. The 16S rRNA, *gyrB*, *rpoB*, and *rpoD* gene sequences of the type strains were retrieved from our previous publications (Mulet et al., 2010, 2012a) and are available in the public National Centre for Biotechnology Information (NCBI) database. A series of individual trees was generated from the 16S rRNA, *gyrB*, *rpoB*, and *rpoD* partial gene sequences. Concatenated gene trees were constructed using the individual alignments in the following order: 16S rRNA (1309 nt), *gyrB* (803 nt), *rpoD* (791 nt), and *rpoB* (923 nt).

The alignments were conducted using a hierarchical method for multiple alignments implemented in the program CLUSTAL_X (Thompson et al., 1997). Automatically aligned sequences were checked manually. Similarities and evolutionary distances were calculated with programs implemented in PHYLIP (Phylogeny Inference Package, version 3.5c) (Felsenstein, 1981). Gene distances were calculated from nucleotide sequences using the Jukes-Cantor method (Jukes and Cantor, 1969), and dendrograms were generated using the neighbor-joining (NJ), minimum-evolution (ME), and maximum parsimony (MP) methods. A bootstrap analysis of 1000 replications was also performed. Values higher than 50% (from 1000) are indicated only at the groups or subgroups branching nodes of the corresponding trees. The topologies of the trees were visualized using the TreeView program (Page, 1996).

### Whole-genome comparisons

Among the *Pseudomonas* genomes, the correlation of the tetranucleotide signatures (TETRA), the average nucleotide identity (ANI) and the GGDC were calculated between pairwise genomic comparisons. The statistical calculations of the tetranucleotide frequencies (TETRA) (Teeling et al., 2004), the ANIb and the ANIm were calculated using the JSpecies software tool available at the webpage http://www.imedea.uib.es/jspecies. To calculate the ANI, the genomic sequence from one of the genomes in a pair (“the query”) was cut by the software into 1020 nucleotide consecutive fragments. The 1020 nt fragments were then used to search against the whole genomic sequence of the other genome in the pair (“the reference”) using the BLASTN algorithm (Altschul et al., 1997). The ANI between the query genome and the reference genome was calculated as the mean identity of all the BLASTN matches that showed more than 30% overall sequence identity over an alignable region of at least 70% of their length. The ANI was calculated based on the BLAST algorithm, ANIb (Altschul et al., 1997; Goris et al., 2007), and the MUMmer ultra-rapid aligning tool, ANIm (Kurtz et al., 2004). The recommended species cut-off was 95% for the ANIb and ANIm indices, and higher than 0.99 for the TETRA signature (Richter and Rosselló-Móra, 2009). The GGDC method functions based on the principle that two genomes are locally aligned using BLAST, which produces a set of high-scoring segment pairs (HSPs); the information in these HSPs is transformed to a single GGDC value using a specific distance formula that sets the species cut-off at 70% similarity. GGDC was calculated using the web service http://ggdc.dsmz.de (Meier-Kolthoff et al., 2013). GGDC 2.0 is an updated and enhanced version with improved DDH-prediction models and additional features such as confidence-interval estimation. The matrices obtained in our study for each parameter were used to generate a dendrogram using Permut Matrix software by applying an average linkage method (UPGMA hierarchical clustering) and Pearson's distance correlation (Caraux and Pinloche, 2005). The dendrograms were constructed using the average value of the duplicate analyses for each strain to assess topology coherence.

### Species delineation based on MLSA and genome indices thresholds

Parametric correlations based on the Pearson's product-moment coefficient and non-parametric correlations using the Spearman's rank correlation coefficient and Kendall tau rank correlation coefficient were calculated between all the whole-genome comparison results and the concatenated phylogenetic MLSA. Correlation analysis were performed using SPSS Plot v.11.0 software. Representations between the whole-genome comparison results and the concatenated phylogenetic MLSA were also graphed.

## Results

### MLSA phylogenetic analysis

The phylogenetic analysis included the 141 reference strains of species that were validly described and used in a previous paper (Mulet et al., 2012a) combined with 112 *Pseudomonas* strains that had complete or draft genomes available in databases (Supplementary Table 1). A series of individual and concatenated phylogenetic trees from 16S rRNA, *gyrB*, *rpoB*, and *rpoD* partial gene alignments were generated. Individual dendrograms were generated using different methods, namely the NJ, MP, and ME methods. Their topologies were congruent (data not shown), as previously demonstrated by Mulet et al. (2010). Phylogenetic groups (G) and subgroups (SG) were defined by the length and branching order of the concatenated gene tree, as previously proposed (Mulet et al., 2010) and updated in 2012 (Mulet et al., 2012a). The name of the first species described in a group or subgroup was chosen to designate that group or subgroup. The resulting groups were supported by high bootstrap values.

**Figure 1:**
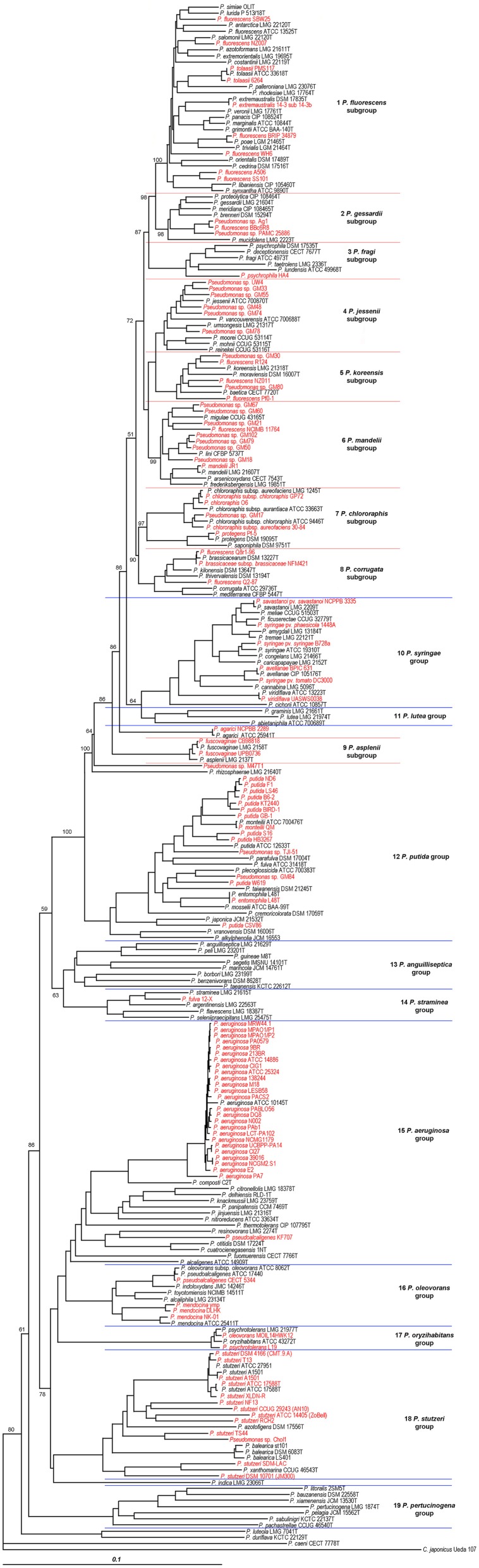
**Phylogenetic tree of the 112 complete or draft genomes of strains related to the genus *Pseudomonas* and the 141 well described *Pseudomonas* strains used in this study based on the phylogenetic analysis of four concatenated genes (16S rRNA, *gyrB*, *rpoB*, and *rpoD*)**. The strains analyzed in this study whose genomes have been sequenced are labeled in red. Distance matrices were calculated by the Jukes-Cantor method. Dendrograms were generated by neighbor-joining. *Cellvibrio japonicum* Ueda107 was used as outgroup. The bar indicates sequence divergence. Percentage bootstrap values only of groups and subgroups higher than 50% of 1000 replicates are indicated at branching nodes.

A phylogenetic tree (Figure 1) was generated based on the concatenated sequences with a total length of 3711 nucleotides in the following order: 16S rRNA (1278 nt), *gyrB* (801 nt), *rpoD* (717 nt), and *rpoB* (915 nt). Phylogenetic assignation to a known species, group or subgroup of the 112 complete or draft genomes analyzed was congruent in all the trees (data not shown). Forty-eight of the 112 strains (42.8% of the genomes analyzed) were located in the same phylogenetic branch as the corresponding species type strain (or genomovar reference strains in the *P. stutzeri* species) with a similarity higher than 97%, which was the accepted species threshold (Mulet et al., 2010, 2012a), and their species assignations were considered correct. As observed in Table 1, 22 of the whole genome sequenced strains studied (20%) were only assigned to the genus and 34 strains (30%) were not correctly identified at the species level. For example, strain BBc6R8, which had been identified as *P. fluorescens*, was included in the *P. gessardii* phylogenetic subgroup, and *P. fulva* 12-X was closer to *P. straminea* than to the *P. fulva* type strain. The closest species type strain for the 112 complete or draft genomes analyzed based on the concatenated analysis of four genes is listed in Table 1 and in Supplementary Table 2. Thirty-seven of the genomes were less than 97% similar to the closest type strain and might be considered representatives of *Pseudomonas* species not yet described or have to be assigned to genomovars in the case of *P. stutzeri*.

**Table 1:** **Phylogenetic affiliation based on concatenated MLSA analysis for the 63 whole genome sequenced strains not assigned, or incorrectly assigned at the species level, including strains of *P. stutzeri* genomovars**.

**Species**	**MLSA similarity %**	**Closest species type strain**	**MLSA similarity %**	**Closest-related strain**	**MLSA similarity % with representative species of the group**	**Representative species of G or SG**	**Group or subgroup**
*P. fluorescens* WH6	96.17	*P. azotoformans* LMG 21611^T^	96.17	*P. azotoformans* LMG 21611^T^	95.03	*P. fluorescens* ATCC 13525^T^	*P. fluorescens* SG/*P. fluorescens* G
*P. fluorescens* SS101	96.11	*P. azotoformans* LMG 21611^T^	98.55	*P. fluorescens* A506	95.11		
*P. fluorescens* A506	96.00	*P. azotoformans* LMG 21611^T^	98.55	*P. fluorescens* SS101	95.03		
*P. fluorescens* BRIP3487	99.54	*P. poae* LGM 21465^T^	99.54	*P. poae* LMG 21465^T^	95.03		
*P. fluorescens* NZ007	98.47	*P. salomonii* LMG 22120^T^	98.47	*P. salomonii* LMG 22120^T^	96.26		
*P. fluorescens* SBW25	96.72	*P. lurida* P 513/18^T^	96.72	*P. lurida* P 513/18^T^	96.07		
*Pseudomonas* sp. Ag1	96.24	*P. brenneri* DSM 15294^T^	99.65	*P. fluorescens* BBc6R8	96.12	*P. gessardii* CIP 105469^T^	*P. gessardii* SG/*P. fluorescens* G
*P. fluorescens* BBc6R8	96.29	*P. brenneri* DSM 15294^T^	99.65	*Pseudomonas* sp. Ag1	96.12		
*Pseudomonas* sp. PAMC25886	96.41	*P. brenneri* DSM 15294^T^	98.36	*P. fluorescens* BBc6R8	95.98		
*P. psychrophila* HA4	95.17	*P. psychrophila* DSM 17535^T^	95.17	*P. psychrophila* DSM 17535^T^	95.10	*P. fragi* ATCC 4973^T^	*P. fragi* SG/*P. fluorescens* G
*Pseudomonas* sp. UW4	97.89	*P. jessenii* ATCC 700870^T^	98.72	*Pseudomonas* sp. GM33	97.89	*P. jessenii* ATCC 700870^T^	*P. jessenii* SG/*P. fluorescens* G
*Pseudomonas* sp. GM48	97.94	*P. jessenii* ATCC 700870^T^	98.36	*Pseudomonas* sp. UW4	97.94		
*Pseudomonas* sp. GM74	97.66	*P. jessenii* ATCC 700870^T^	97.70	*Pseudomonas* sp. GM48	97.66		
*Pseudomonas* sp. GM55	97.94	*P. jessenii* ATCC 700870^T^	98.00	*Pseudomonas* sp. GM33	97.94		
*Pseudomonas* sp. GM33	97.97	*P. jessenii* ATCC 700870^T^	98.72	*Pseudomonas* sp. UW4	97.97		
*Pseudomonas* sp. GM78	98.58	*P. umsongensis* LMG 21317^T^	98.58	*P. umsongensis* LMG 21317^T^	96.91		
*P. fluorescens* Pf0-1	96.46	*P. koreensis* LMG 21318^T^	96.55	*Pseudomonas* sp. GM30	96.46	*P. koreensis* LMG 21318^T^	*P. koreensis* SG/*P. fluorescens* G
*Pseudomonas* sp. GM30	97.78	*P. koreensis* LMG 21318^T^	98.33	*P. fluorescens* R124	97.78		
*P. fluorescens* R124	97.53	*P. koreensis* LMG 21318^T^	98.33	*Pseudomonas* sp. GM30	97.53		
*P. fluorescens* NZ011	96.80	*P. koreensis* LMG 21318^T^	97.11	*Pseudomonas* sp. GM80	96.80		
*Pseudomonas* sp. GM80	96.66	*P. baetica* CECT 7720^T^	97.11	*P. fluorescens* NZ011	95.97		
*Pseudomonas* sp. GM50	98.58	*P. lini* CFBP 5737^T^	99.51	*Pseudomonas* sp. GM102	96.83	*P. mandelii* LMG 21607^T^	*P. mandelii* SG/*P. fluorescens* G
*Pseudomonas* sp. GM18	97.67	*P. lini* CFBP 5737^T^	97.84	*Pseudomonas* sp. GM50	96.49		
*Pseudomonas* sp. GM60	97.14	*P. migulae* CCUG 43165^T^	99.35	*Pseudomonas* sp. GM67	96.06		
*Pseudomonas* sp. GM21	97.36	*P. lini* CFBP 5737^T^	97.36	*P. lini* CFBP 5737^T^	96.94		
*P. fluorescens* NCIMB 11764	96.73	*P. migulae* CCUG 43165^T^/*P. lini* CFBP 5737^T^	96.96	*Pseudomonas* sp. GM21	96.38		
*Pseudomonas* sp. GM79	98.47	*P. lini* CFBP 5737^T^	99.54	*Pseudomonas* sp. GM102	96.71		
*Pseudomonas* sp. GM102	98.53	*P. lini* CFBP 5737^T^	99.54	*Pseudomonas* sp. GM79	96.80		
*Pseudomonas* sp. GM67	97.42	*P. migulae* CCUG 43165^T^	99.35	*Pseudomonas* sp. GM60	96.38		
*Pseudomonas* sp. GM17	99.02	*P. chlororaphis* subsp. *chlororaphis* ATCC 9446^T^	99.05	*P. chlororaphis* subsp. *aureofaciens* 30-84	99.02	*P. chlororaphis* subsp. *chlororaphis* ATCC 9446^T^	*P. chlororaphis* SG/*P. fluorescens* G
*P. chlororaphis* subsp. *aureofaciens* 30-84	98.83	*P. chlororaphis* subsp. *chlororaphis* ATCC 9446^T^	99.05	*Pseudomonas* sp. GM17	98.83		
*P. chlororaphis* 06	99.65	*P. chlororaphis* subsp. *aureofaciens* LMG 1245^T^	99.73	*P. chlororaphis* subsp. *chlororaphis* PG72	98.16		
*P. chlororaphis* subsp. *chlororaphis* PG72	99.86	*P. chlororaphis* subsp. *aureofaciens* LMG 1245^T^	99.86	*P. chlororaphis* subsp. *aureofaciens* LMG 1245^T^	98.00		
*P. fluorescens* Q8r1-96	99.81	*P. brassicacearum* DSM 13227^T^	99.92	*Pseudomonas brassicaceae* subsp. *brassicaceae* NFM 421	95.23	*P. corrugata* ATCC 29736^T^	*P. corrugata* SG/*P. fluorescens* G
*P. fluorescens* Q2-87	96.88	*P. brassicacearum* DSM 13227^T^	97.05	*Pseudomonas brassicaceae* subsp. *brassicaceae* NFM 421	95.03		
*Pseudomonas* sp. M47T1	91.01	*P. koreensis* LMG 21318^T^	91.04	*P. fluorescens* R124	–	–	n.a. SG*/P. fluorescens* G
*P. syringae* pv. *phaesicola* 1448A	99.02	*P. ficuserectae* CCUG 32779^T^	99.02	*P. ficuserectae* CCUG 32779^T^	96.18	*P. syringae* ATCC 19310^T^	*P. syringae* G
*P. syringae* pv. *tomato* DC3000	98.78	*P. avellanae* CIP 105176^T^	98.78	*P. avellanae* BPIC 631	94.66		
*P. putida* KT2440	96.80	*P. monteilii* ATCC 700476^T^	99.35	*P. putida* BIRD-1	95.44	*P. putida* ATCC 12633^T^	*P. putida* G
*P. putida* W619	94.78	*P. plecoglossicida* ATCC 700383^T^	95.33	*Pseudomonas* sp. GM84	93.64		
*P. putida* GB-1	96.80	*P. monteilii* ATCC 700476^T^	97.09	*P. putida* KT2440	95.93		
*P. putida* S16	96.04	*P. monteilii* ATCC 700476^T^	98.47	*P. putida* HB3267	95.10		
*P. putida* CSV86	94.97	*P. japonica* JCM 21532^T^	94.97	*P. japonica* JCM 21532^T^	90.32		
*P. putida* HB3267	96.44	*P. monteilii* ATCC 700476^T^	98.47	*P. putida* S16	95.41		
*P. putida* BIRD-1	96.61	*P. monteilii* ATCC 700476^T^	99.35	*P. putida* KT2440	95.13		
*Pseudomonas* sp. TJI-51	94.81	*P. monteilii* ATCC 700476^T^	94.92	*P. putida* HB3267	94.11		
*Pseudomonas* sp. GM84	96.47	*P. plecoglossicida* ATCC 700383^T^	96.47	*P. plecoglossicida* ATCC 700383^T^	93.58		
*P. putida* B6-2	96.80	*P. monteilii* ATCC 700476^T^	99.65	*P. putida* ND6	95.41		
*P. putida* ND6	96.80	*P. monteilii* ATCC 700476^T^	99.87	*P. putida* F1	95.50		
*P. putida* F1	96.72	*P. monteilii* ATCC 700476^T^	99.87	*P. putida* ND6	95.41		
*P. putida* LS46	96.89	*P. monteilii* ATCC 700476^T^	99.68	*P. putida* ND6	95.38		
*P. fulva* 12-X	96.47	*P. straminea* LMG 21615^T^	96.47	*P. straminea* LMG 21615^T^	96.47	*P. straminea* LMG 21615^T^	*P. straminea* G
*P. pseudoalcaligenes* KF707	91.63	*P. citronellolis* LMG 18378^T^	91.63	*P. citronellolis* LMG 18378^T^	88.67	*P. aeruginosa* ATCC10145^T^	*P. aeruginosa* G
*P. pseudoalcaligenes* CECT 5344	99.81	*P. oleovorans* subsp. *oleovorans* ATCC 8062^T^	99.81	*P. oleovorans* subsp. *oleovorans* ATCC 8062^T^	99.81	*P. oleovorans* subsp. *oleovorans* ATCC 8062^T^	*P. oleovorans* G
*P. oleovorans* MOIL14HWK12	99.54	*P. psychrotolerans* LMG 21977^T^	99.54	*P. psychrotolerans* LMG 21977^T^	99.40	*P. oryzihabitans* ATCC 43272^T^	*P. oryzihabitans* G
*P. stutzeri* NF13	92.57	*P. stutzeri* ATCC 17588^T^	93.28	*P. stutzeri* CCUG 29243	92.57	*P. stutzeri* ATCC 17588^T^	*P. stutzeri* G
*P. stutzeri* SDM-LAC	94.81	*P. xanthomarina* CCUG 45643^T^	94.81	*P. xanthomarina* CCUG 45643^T^	88.72		
*P. stutzeri* DSM 10701 (JM300)	90.45	*P. stutzeri* ATCC 17588^T^	91.27	*P. stutzeri* TS44	90.45		
*P. stutzeri* ATCC 14445 (ZoBell)	90.55	*P. stutzeri* ATCC 17588^T^	92.27	*P. stutzeri* RCH2	90.55		
*P. stutzeri* RCH2	92.54	*P. stutzeri* ATCC 17588^T^	92.87	*P. stutzeri* CCUG 29243	92.54		
*P. stutzeri* TS44	92.06	*P. stutzeri* ATCC 17588^T^	95.18	*Pseudomonas* sp. Chol1	92.06		
*P. stutzeri* CCUG 29243 (AN10)	91.34	*P. stutzeri* ATCC 17588^T^	93.28	*P. stutzeri* NF13	91.34		
*Pseudomonas* sp. Chol1	91.94	*P. stutzeri* ATCC 17588^T^	95.18	*P. stutzeri* TS44	91.94		

*n.a., not assigned*.

### Genomic analysis and correlation with MLSA

TETRA, average nucleotide identity based on MUMmer (ANIm) or BLAST (ANIb) and GGDC comparisons were calculated for the 112 genomes. In total, each final dataset consisted of 12,544 pairwise values, including the 112 pairwise comparisons of each genome with itself. Each square matrix obtained was transformed in a lower-triangular matrix using the average value of the duplicate analyses for each pair. The final dataset consisted of 6328 pairwise values. A dendrogram was generated for each matrix to assess their phylogenetic coherence (data not shown). The ANIb dendrogram showed the best topology congruence compared to the MLSA phylogenetic tree (Supplementary Figure 1).

**Figure 2:**
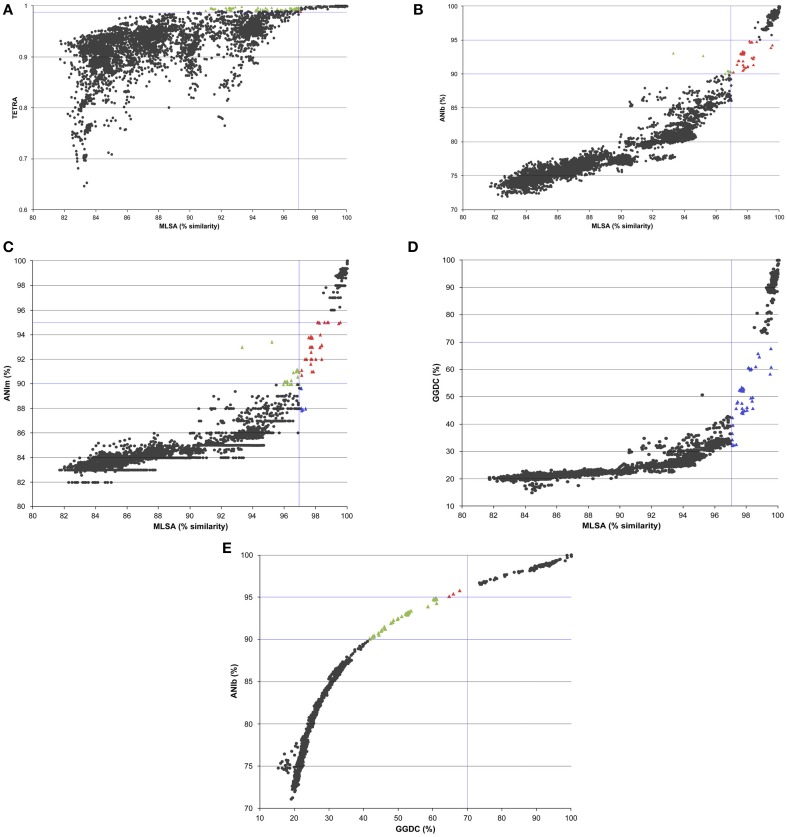
**Graphs representing the relationship between TETRA (A), ANIb (B), ANIm (C), and GGDC (D) indices vs. MLSA sequence similarity for the genomes studied; (E) shows the relationship between ANIb and GGDC indices**. Each dot represents a pairwise comparison; the genomic indices are plotted against the corresponding MLSA sequence similarity. TETRA signatures values in black circles indicate TETRA < 0.99 and MLSA < 97% and TETRA > 0.99 and MLSA > 97%; green triangles indicate TETRA > 0.99 and MLSA < 97%; and red triangles TETRA < 0.99 and MLSA > 97%. ANIb and ANIm black circles indicate genomic values <90% and MLSA < 97% and genomic values >95% and MLSA > 97%; green triangles genomic values between 90 and 95% and MLSA < 97%; red triangles genomic values between 90 and 95% and MLSA > 97%; and blue circles genomic values between 85 and 90% and MLSA > 97%. The values of GGDC < 70% and MLSA < 97% and GGDC > 70% and MLSA > 97% are indicated in black circles in GGDC plots; in green triangles are indicated values of GGDC < 70% and MLSA > 97%.

**Figure 3:**
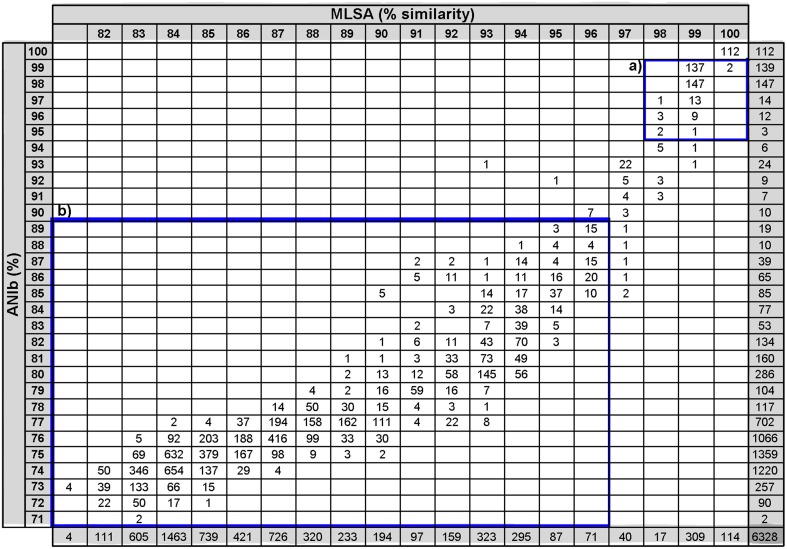
**Association table between ANIb values and MLSA sequence similarities**. The number of strain pairs is displayed in each category square. Square A indicates ANIb values ≥95% and MLSA ≥97%; square B indicates ANIb values <90% and MLSA values <97%.

Phylogenetic similarities in the analysis of the four concatenated genes were compared with all the indices calculated in the whole genome analyses. The results are plotted in Figure 2. The overall relationship between the MLSA analysis and the whole-genome analysis was found to be non-linear, which is consistent with previous studies performed using ANI and 16S rRNA gene sequence similarities (Konstantinidis and Tiedje, 2005a,b; Mulet et al., 2010; Kim et al., 2014). Correlation analyses between all the whole-genome comparison results and concatenated phylogenetic MLSA distances were also performed (Supplementary Table 3). Among the various methods proposed for substituting the experimental DDH high correlations were found between MLSA and ANIb (0.933 Spearman's rho and 0.917 Pearson coefficients) and between MLSA and GGDC (0.928 Spearman's rho and 0.766 Pearson's coefficients). The Pearson's correlation coefficients between ANIm and MLSA, GGDC and MLSA, and TETRA and MLSA were 0.838, 0.766, and 0.65, respectively. The ANIb values were selected and thoroughly analyzed as indicated in Figure 3. Square A (ANIb ≥ 95; MLSA ≥ 97, the threshold species delimitation by each index) included 315 pairwise comparisons of strains of the same species, such as *P. aeruginosa*, *P. mendocina*, and *P. putida*, subspecies of *P. oleovorans*, and *P. stutzeri* members of the same genomovar. Most of the values (5845) accumulated in square B (ANIb < 90%; MLSA < 97%) and corresponded to interspecies comparisons. In a few cases, strains in the pairwise comparisons located in square A were assigned to different species, which supported a non-correct species identification for at least one of the strains compared (i.e., the pair *P. brassicacearum* NFM 421 and *P. fluorescens* Q8r1 or the pair *P. oleovorans* MOIL14HWK12 and *P. psychrotolerans* L19). The transition between squares A and B (ANIb in the range of <95% and ≥90%) included only 56 (0.9%) pairwise comparisons of strains; nine of those comparisons had MLSA values <97% and 47 comparisons had MLSA values ≥97%. Twenty-four of the 56 pair-wise values were combinations between *P. aeruginosa* PA7 (considered a taxonomic outlier of the *P. aeruginosa* species; Roy et al., 2010) and the other genomes of the *P. aeruginosa* strains. The rest of the values (32) were distributed among a few strains in the *P. chlororaphis* SG (three strains, two comparisons), the *P. fluorescens* SG (two strains, one comparison), the *P. gessardii* SG (two strains, one comparison), the *P. jessenii* SG (five strains, nine comparisons), the *P. mandelii* SG (seven strains, seven comparisons), the *P. putida* G (seven strains, six comparisons) and the *P. stutzeri* SG (four strains, two comparisons). These last two pairs of *P. stutzeri* strains were clearly outliers in the plot: AN10/NF13 (MLSA 93% and ANIb 93%) and Chol1/TS44 (MLSA 95% and ANIb 92%). The four strains had been phylogenetically assigned to four different genomovars of *P. stutzeri* (Lalucat et al., 2006; Peña et al., 2013).

Six pairwise values were located in the region of 97% MLSA similarity and below 90% ANIb and corresponded to two strains in the *P. corrugata* SG (classified as *P*. *fluorescens* Q2-87 and *P. brassicacearum* subsp. *brassicacearum* NFM421), three strains (*P. fluorescens* R124, *P. fluorescens* NZ011, and *Pseudomonas* sp. GM80) were in the *P. koreensis* SG, and four strains were in the *P. mandelii* SG (*Pseudomonas* sp. GM21, GM50, GM79, and GM102).

The graph representations for ANIm, ANIb, and GGDC plotted against MLSA (Figure 2) were very similar although the proposed species threshold for GGDC (70%) appeared to clearly discriminate the species boundaries because only a few pairs of strains (36 comparisons) were found between 50 and 70% GGDC distances, including combinations of the same strains as those detected in the ANIb/MLSA comparisons (strain PA7, strains in the *P. chlororaphis* SG and *P. mandelii* SG and four strains in the *P. stutzeri* G). In an attempt to find discontinuities in the graphs, the ANIb values were plotted against the GGDC values (Figure 2E). A high correlation of 0.940 was found between ANIb and GGDC, and a discontinuity might be observed in the region with 94–96% ANIb and 52–70% GGDC, in which only nine comparisons were detected and can be considered exceptions. Each pair of strains was closely related in the MLSA phylogenetic tree: *P. avellanae* 631/*P*. *syringae* DC300; *Pseudomonas* sp. GM50/GM102; *Pseudomonas* sp. GM33/*P. putida* UW4; *P. chlororaphis* strains; *P. fluorescens* 86/A506; and *P. fluorescens* A506/SS101.

**Figure 4:**
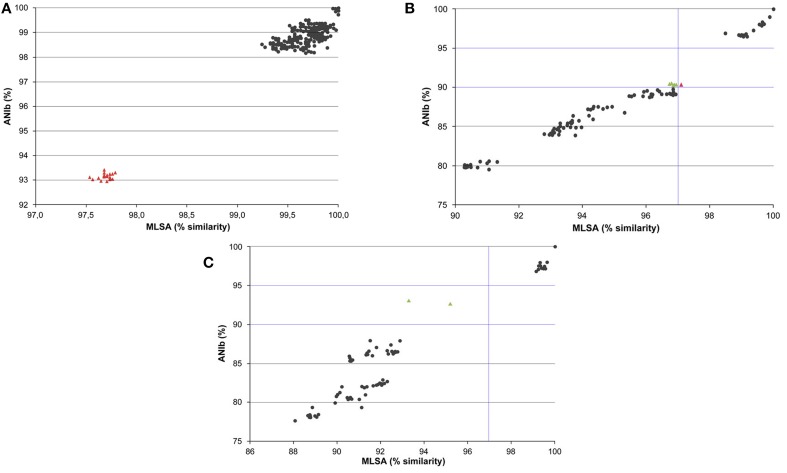
**Graphs representing relationships between ANIb values and MLSA sequence similarities of pairwise comparisons of strains assigned to the *P. aeruginosa* (A), *P. putida* (B), and *P. stutzeri* (C) groups**. Each dot represents pairwise values between the ANIb indices plotted against the corresponding MLSA sequence similarity. In the ANIb range of 90–95%, green triangles indicate pairwise comparisons with MLSA values lower than 97% and red triangles values between 97 and 98%; other values are indicated with black circles.

Relationships between the MLSA and ANIb values were analyzed independently for the genomes of species in the *P. aeruginosa*, *P. putida*, and *P. stutzeri* phylogenetic groups (Figure 4). Correlation indices are shown in Supplementary Table 4. High Pearson's correlation coefficients between the MLSA and ANIb indices were detected among the strains in these three phylogenetic groups (i.e., 0.976, 0.983, and 0.971, respectively). In the three graphs, two different clusters were observed. In the *P. aeruginosa* group plot, a cluster at MLSA similarities higher than 98% and ANIb values higher than 98% was observed, and another cluster showed MLSA similarities of approximately 97.5% and ANIb values of approximately 93%. This last cluster of red-colored triangles in Figure 4A included all pairwise value combinations of *P. aeruginosa* PA7 with the other *P. aeruginosa* strains. In the *P. putida* and *P. stutzeri* graphs, the first cluster belonged to MLSA similarities higher than 97% and ANIb values higher than 95%, corresponding to intraspecies and intragenomovar comparisons, whereas a second cluster had MLSA similarities lower than 97% and ANIb values lower than 91%. Clear gaps were observed in the three plots.

## Discussion

Bacterial species are considered groups of strains that are characterized by a certain degree of phenotypic consistency, by a significant degree (70%) of DNA–DNA hybridisation (DDH; Wayne et al., 1987) and by over 98.7–99% of 16S ribosomal RNA gene sequence similarity (Stackebrandt and Ebers, 2006). 16S rRNA gene sequences are highly conserved among strains of the same bacterial species and are frequently used to identify and classify microorganisms. Taxonomic classifications based only on the analysis of the 16S rRNA gene can create misclassifications in some instances, and the additional analysis of other housekeeping gene sequences should be performed for a correct phylogenetic affiliation. A threshold of 97% similarity in the MLSA study of four housekeeping genes (16S rRNA, *gyrB*, *rpoD*, and *rpoB* genes) has been proposed by Mulet et al. (2010, 2012a) for species differentiation in the genus *Pseudomonas*. The whole genome analysis of 112 *Pseudomonas* strains in the present study revealed that these genomes were in the range of 71–99% ANIb values, resulting in a continuous gradient of genetic relatedness, as shown in Figure 3. This type of continuous genetic gradient has been previously reported for the 10 strains studied by Konstantinidis et al. (2009) in the *Shewanella* genus. However, only a few strain comparisons were found in the ANIb region between 90 and 95% or in the GGDC region between 50 and 70%, which are below the species thresholds. The strains in these regions were classified as subspecies or genomovars within a species or were considered taxonomic outliers. The ANIb and GGDC values between these strains were consistent with the experimental DDH in the *P. stutzeri* group (Lalucat et al., 2006), the *P. putida* group (Regenhardt et al., 2002), and the *P. aeruginosa* group (Roy et al., 2010). These pairs of strains are always phylogenetically closely related, and extensive genetic exchange between each pair, or with other bacteria of the group or of other groups cannot be excluded. These strains can be considered different ecotypes of the same species or strains in the speciation process. A comparative study of their genomes must be conducted to confirm this possibility, which has been performed for the *P. aeruginosa* strain PA7. This strain is considered an outlier within the species, and its genome contains a similar number of genes as the other *P. aeruginosa* strains, but more than 1000 exclusive genes were found in PA7 compared with the *P. aeruginosa* strains PAO1, PA14, and LESB58 (Roy et al., 2010). This is a good example of the difficulty in obtaining taxonomic conclusions until the whole genome sequence of all type strains of the species in the group are available.

Intraspecies comparisons were characterized by ANIb values higher than 95%, which corresponds to MLSA values higher than 97% and GGDC values higher than 70%; these thresholds are considered the species boundaries by the three methods. However, the strains in the *P. putida* G assigned to the *P. putida* species were genomically very diverse, and some of those strains likely represent a new species, as has been proposed by Mulet et al. (2013) and more recently by Ohji et al. (2014).

*P. aeruginosa*, the type species of the genus, is phenotypically represented by homogeneous strains. Generally, there is no doubt in the identification of this species, and the strains studied were also very coherent in the genomic comparisons. However, this is not always true for other *Pseudomonas* species. The analysis of the 112 draft or complete genomes revealed that 63 strains (57%) were not assigned to species (22 strains) or were not correctly assigned (34 strains). These data were supported by the MLSA analysis and the whole-genome comparisons based on ANIb and GGDC. This result raised the question of which species those 56 genomes belonged to. The correct identification at the species level requires a polyphasic taxonomic study, but the MLSA tree, which included all the type strains, provides an adequate phylogenetic assignation to known species or the prediction of putative novel species. It is important to emphasize that incorrect identifications can lead to mistaken conclusions.

Several recent studies support our observation of wrong assignation to species of strains in the genus *Pseudomonas*. Duan and collaborators revealed that *Pseudomonas* sp. UW4 belongs to the *fluorescens* group, specifically the *P. jessenii* subgroup, and not to *P. putida* as previously proposed (Duan et al., 2013). Paulsen et al. (2005) published the complete genome of *P. fluorescens* Pf-5, and this strain was later reclassified as *P. protegens* Pf-5 (Ramette et al., 2011).

MLSA is the most convenient method nowadays for the assessment of the phylogenetic relationships among the species in the genus *Pseudomonas* until whole genome sequences of the type strains are available, and the best correlation with MLSA was found with ANIb in the study of the different digital whole genome comparisons tested. However, the use of GGDC was shown to be useful in species discrimination. As previously observed by other authors, most of the intraspecies ANIb values were found to be higher than 96%, which is within the range previously recommended for species delineation (Konstantinidis and Tiedje, 2005a,b) and corresponds to MLSA values higher than 97%, as proposed by Mulet et al. (2010).

In conclusion, because the resolution of the 16S rRNA tree was not sufficient to differentiate 63 genomes from other closely related *Pseudomonas* species, the classification of these bacteria should follow the phylogeny of the housekeeping genes until the whole genome sequence of the type strains of all *Pseudomonas* species is known. In recent years, thanks to NGS technologies, a remarkable increase in the number of sequenced genomes, drafts or complete, are available, but the correct assignation of the sequenced strains to the corresponding species with the accepted taxonomic tools is important before comparative analyses with other genomes can be performed. The need for the whole genome sequences of all the type strains, which are the only species references that are publicly available in culture collections, is evident. The project “Genomic Encyclopedia of Bacteria and Archaea: Sequencing a Myriad of Type Strains” (GEBA project) was initiated to address this problem (Kyrpides et al., 2014).

### Conflict of interest statement

The authors declare that the research was conducted in the absence of any commercial or financial relationships that could be construed as a potential conflict of interest.
