# Supplementary material for: Phylogenomics and systematics in Pseudomonas
Source: Front Microbiol. 2015 Mar 18;6:214. doi: 10.3389/fmicb.2015.00214 (PMC4447124; doi:10.3389/fmicb.2015.00214)
Supplement: Supplementary file 1 [file Table1.PDF]

Table S1. List of the 112 draft or complete genomes analyzed with the genome accession number, number of contigs and origin.

Genomes	Status	Genome Accession Number	Number of Contigs	Origin
P. aeruginosa DQ8	Draft	ALIO01000001-ALIO01000376	376	Soil
P. aeruginosa LCT-PA102	Draft	AJKG01000001-AJKG01000180	180	Clinical
P. aeruginosa 138244	Draft	AEVV00000001 - AEVV00000381	381	Clinical
P. aeruginosa 14886	Draft	AKZD01000001-AKZD01000211	211	Soil
P. aeruginosa 213BR	Complete	AFXK01000001	1	Clinical
P. aeruginosa ATCC 25324	Draft	AKZE01000001-AKZE01001023	1023	Environmental (Glass crusher air plate)
P. aeruginosa 39016	Complete	AEEX01000001-AEEX01000349	349	Clinical
P. aeruginosa 9BR	Complete	AFXI01000001-AFXI01000002	2	Clinical
P. aeruginosa CI27	Draft	AKZG01000001-AKZG01000160	160	Clinical
P. aeruginosa CIG1	Draft	AKBD01000001-AKBD01000544	544	Clinical
P. aeruginosa E2	Draft	AKZH01000001-AKZH01000196	196	Tomato plant
P. aeruginosa LESB58	Complete	NC_011770	1	Clinical
P. aeruginosa M18	Complete	NC_017548	1	Rhizosphere
P. aeruginosa MPAO1	Draft	AHKM01000001-AHKM01000140	140	Clinical
P. aeruginosa MRW44.1	Draft	ALBW01000001-ALBW01000054	54	Laboratory evolved inducible mutator isolate
P. aeruginosa N002	Draft	ALBV02000001-ALBV02000235	235	Soil
P. aeruginosa NCGM2.S1	Complete	NC_017549.1	1	Clinical
P. aeruginosa NCMG1179	Draft	BADP01000001-BADP01000290	219	Clinical
P. aeruginosa PA0579	Draft	ALOF01000001-ALOF01000016	16	Clinical
P. aeruginosa PA7	Complete	NC_009656	147	Clinical
P. aeruginosa PAb1	Draft	ABKZ01000001-ABKZ01000658	658	Clinical
P. aeruginosa PABLO56	Draft	ALPS01000001-ALPS01000401	401	Clinical
P. aeruginosa PACS2	Complete	AAQW01000001	1	Clinical
P. aeruginosa PAO1	Complete	NC_002516	1	Clinical
P. aeruginosa UCBPP-PA14	Complete	NC_008463	1	Clinical
P. agarici NCBPP 2289	Draft	AKBQ01000001-AKBQ01000699	699	Mushroom pathogenic pseudomonads
P. avellanae BPIC 631	Draft	AKBS01000001-AKBS01001602	1602	Host
P. brassicacearum subsp. *brassicacearum* NFM421	Complete	NC_015379	1	Laboratory strain
P. chlororaphis subsp. *aureofaciens* 30-84	Complete	AHHJ01000001-AHHJ01000013	13	A root of wheat

P. chlororaphis subsp. *chlororaphis* GP72	Draft	AHAY01000001-AHAY01000347	347	Green pepper rhizosphere
P. chlororaphis O6	Complete	AHOT01000001-AHOT01000030	1	Soil
P. entomophila L48	Complete	CT573326.1	1	Pathogen
P. extremaustralis 14-3 substr. 14-3b	Draft	AHIP01000001-AHIP01000135	135	Temporary water pond in Antarctica
P. fluorescens Pf-01	Complete	CP000094.2	1	Agricultural loam Soil
P. fluorescens NCIMB 11764	Complete	ALWP02000001-ALWP02000145	145	Enrichment culture-supplied potassium cyanide
P. fluorescens BRIP34879	Draft	AMZW01000001-AMZW01000261	261	Cereal Crops
P. fluorescens A506	Complete	CP003041.1	1	Leaf of a pear tree
P. fluorescens BBc6R8	Draft	AKXH02000001-AKXH02000153	153	Sporocarp of *Laccaria bicolor* strain S238N in a Douglas fir plantation
P. fluorescens Q2-87	Complete	AGBM01000001-AGBM01000002	2	Roots of wheat
P. fluorescens Q8r1-96	Complete	AHPO01000001-AHPO01000005	4	Roots of wheat
P. fluorescens R124	Complete	ALYL01000001-ALYL01000078	78	Orthoquartzite cave surface
P. fluorescens SBW25	Complete	AM181176.4	1	Leaf surfaces of a sugar beet plant
P. fluorescens SS101	Complete	AHPN01000001	1	Rhizosphere of wheat
P. fluorescens NZ011	Draft	AJXJ01000001-AJXJ01000973	973	Unknown strain (pathogen)
P. fluorescens WH6	Complete	AEAZ01000001-AEAZ01000053	53	Wheat rhizosphere
P. fluorescens NZ007	Draft	AKBR01000001-AKBR01000809	809	Pathogen
P. fulva 12-X	Complete	CP002727.1	1	Rice paddy
P. fuscovaginae CB98818	Draft	ALAQ01000001-ALAQ01000261	261	Pathogenic to rice sheath
P. fuscovaginae UPB0736	Draft	AIEU01000001-AIEU01000273	273	Broad host range plant-pathogen
P. mandelii JR1	Draft	AJFM01000001-AJFM01000096	96	Natural mineral waters
P. mendocina DLHK	Draft	ALKM01000001-ALKM01000033	33	Biotrickling reactor
P. mendocina NK-01	Complete	CP002620.1	1	Farmland Soil
P. mendocina ymp	Complete	CP000680.1	1	Sediment, surface holding pond
P. monteilii QM	Draft	AHGZ01000001-AHGZ01000278	278	Soil
P. oleovorans MOIL14HWK12	Draft	AZOB01000001-AZOB01000019	19	Rhizosphere
P. protegens Pf-5	Complete	CP000076.1	1	Plant commensal
P. pseudoalcaligenes CECT 5344	Draft	CAIG01000001-CAIG01000024	24	Cyanide-degrading bacterium
P. pseudoalcaligenes KF707	Draft	AJMR01000001-AJMR01000233	233	Soil polychlorinated biphenyl (PCB) degrader
P. putida B6-2	Draft	AGCS01000001-AGCS01000027	27	Soil
P. putida BIRD-1	Complete	NC_017530	1	Rhizosphere
P. putida CSV86	Draft	AMWJ01000001-AMWJ01000209	209	Soil isolate

P. putida F1	Complete	NC_009512	1	Polluted creek
P. putida GB-1	Complete	NC_010322	1	Fresh water
P. putida KT2440	Complete	NC_002947	1	Derived from a toluene-degrading isolate
P. putida LS46	Draft	ALPV02000001-ALPV02000032	32	Enrichment from wastewater
P. putida ND6	Complete	NC_017986	1	Industrial wastewater
P. putida HB3267	Complete	NC_019905	1	Human skin
P. putida S16	Complete	NC_015733	1	Nicotine-degrader rhizosphere
Pseudomonas sp. UW4	Complete	NC_019670	1	Rhizosphere
P. putida W619	Complete	NC_010501	1	Black Cottonwood tree
P. savastonoi pv. *savastanoi* NCPPB 3335	Complete	ADMI02000001-ADMI02000397	397	Olive knot, plant pathogen
Pseudomonas sp. Ag1	Draft	AKVH01000001-AKVH01000113	113	Mosquito gut, after a blood meal
Pseudomonas sp. Chol1	Draft	AMSL01000001-AMSL01000199	199	Soil
Pseudomonas sp. GM102	Draft	AKJB01000001-AKJB01000159	159	Root
Pseudomonas sp. GM17	Draft	AKJU01000001-AKJU01000280	280	Root
Pseudomonas sp. GM18	Draft	AKJT01000001-AKJT01000140	140	Root
Pseudomonas sp. GM21	Draft	AKJS01000001-AKJS01000210	210	Root
Pseudomonas sp. GM30	Draft	AKJP02000001-AKJP02000032	32	Root
Pseudomonas sp. GM33	Draft	AKJO01000001-AKJO01000205	205	Root
Pseudomonas sp. GM48	Draft	AKJM01000001-AKJM01000200	200	Root
Pseudomonas sp. GM50	Draft	AKJK01000001-AKJK01000155	155	Root
Pseudomonas sp. GM55	Draft	AKJJ01000001-AKJJ01000163	163	Root
Pseudomonas sp. GM60	Draft	AKJI01000001-AKJI01000181	181	Root
Pseudomonas sp. GM67	Draft	AKJH01000001-AKJH01000183	183	Root
Pseudomonas sp. GM74	Draft	AKJG01000001-AKJG01000180	180	Root
Pseudomonas sp. GM78	Draft	AKJF01000001-AKJF01000235	235	Root
Pseudomonas sp. GM79	Draft	AKJE01000001-AKJE01000126	126	Root
Pseudomonas sp. GM80	Draft	AKJD01000001-AKJD01000282	282	Root
Pseudomonas sp. GM84	Draft	AKJC01000001-AKJC01000384	384	Root
Pseudomonas sp. M47T1	Draft	AJWX01000001-AJWX01000088	88	*Bursaphelenchus xylophilus* host
Pseudomonas sp. PAMC 25886	Draft	AHHC01000001-AHHC01000095	95	Glacier cryoconite
Pseudomonas sp. TJI-51	Draft	AEWE01000001-AEWE01001069	1069	Wastewater
P. stutzeri ATCC 17588	Complete	NC_015740	1	Clinical
P. stutzeri DSM 4166	Complete	NC_017532	1	Rhizosphere

P. stutzeri A1501	Complete	NC_009434	1	Rice roots
P. stutzeri CCUG 29243 (AN10)	Complete	NC_018028	1	Marine water
P. stutzeri RCH2	Complete	NC_019936	1	Host, Soil, Rice roots
P. stutzeri SDM-LAC	Draft	AGSX01000001-AGSX01000199	199	Host, Marine, Rice roots, Soil
P. stutzeri T13	Draft	ALJB01000001-ALJB01000071	71	Activated sludge
P. stutzeri TS44	Draft	AJXE01000001-AJXE01000078	78	Highly arsenic-contaminated soil from metal (gold, copper, iron) mines
P. stutzeri DSM 10701 (JM300)	Complete	NC_018177	1	Soil
P. stutzeri NF13	Draft	AOBS01000001-AOBS01000082	82	Marine water
P. stutzeri XLDN-R	Draft	AKYE01000001-AKYE01000167	167	Soil samples collected at a site near a petroleum refinery
P. stutzeri ATCC 14405 (ZoBell)	Draft	AGSL01000001-AGSL01000130	130	Marine sample from the Pacific Ocean
P. syringae pv. *phaseolicola* 1448A	Complete	NC_005773	1	*Phaseolus vulgaris*
P. syringae B728a	Complete	NC_007005	1	Plant pathogen
P. psychrotolerans L19	Draft	AHBD01000001-AHBD01000053	53	European 50-cent copper alloy coin
P. syringae pv. *tomato* str. DC3000	Complete	NC_004578	1	Tomato
P. psychrophila HA4	Draft	ALJC01000001-ALJC01000145	145	Activated sludge sample
P. tolaasii 6264	Draft	AKYY01000001-AKYY01000688	688	Parasite
P. tolaasii PSM117	Draft	AJXG01000001-AJXG01000357	357	Mushroom pathogen *P. tolaasii*
P. viridiflava UASWS0038	Draft	AMQP01000001-AMQP01000201	201	Plant Symbiotic
