# Supplementary material for: Phylogenomics and systematics in Pseudomonas
Source: Front Microbiol. 2015 Mar 18;6:214. doi: 10.3389/fmicb.2015.00214 (PMC4447124; doi:10.3389/fmicb.2015.00214)
Supplement: Supplementary file 2 [file Table2.PDF]

Table S2. Phylogenetic affiliation based on MLSA analysis of the 112 draft or complete genome strains analysed in the study.

Species	MLSA similarity %	Closest-related strain	MLSA similarity % with type strain	Representative species	Group or Subgroup
P. fluorescens WH6	96.17	*P. azotoformans* LMG 21611^{T}	95.03	*P. fluorescens* ATCC 13525^{T}	*P. fluorescens* SG / *P. fluorescens* G
P. extremoaustralis 14-3 substrain 14-3b	99.92	*P. extremoaustralis* DSM 17835^{T}	95.29		
P. fluorescens SS101	98.55	*P. fluorescens* A506	95.11		
P. fluorescens A506	98.55	*P. fluorescens* SS101	95.03		
P. fluorescens BRIP 34879	99.54	*P. poae* LGM 21465^{T}	95.03		
P. fluorescens NZ007	98.47	*P. salomonii* LMG 22120^{T}	96.26		
P. tolaasii 6264	99.59	*P. tolaasii* PSM117	94.89		
P. tolaasii PSM117	99.92	*P. tolaasii* ATCC 33618^{T}	94.92		
P. fluorescens SBW25	96.72	*P. lurida* P513/18^{T}	96.07		
Pseudomonas sp. Ag1	99.65	*P. fluorescens* BBc6R8	96.12	*P. gessardii* CIP 105469^{T}	*P. gessardii* SG / *P. fluorescens* G
P. fluorescens BBc6R8	99.65	*Pseudomonas* sp. Ag1	96.12		
Pseudomonas sp. PAMC25886	98.36	*P. fluorescens* BBc6R8	95.98		
P. psychrophila HA4	95.17	*P. psychrophila* DSM 17535^{T}	95.10	*P. fragi* ATCC 4973^{T}	*P. fragi* SG / *P. fluorescens* G
Pseudomonas sp. UW4	98.72	*Pseudomonas* sp. GM33	97.89	*P. jessenii* ATCC 700870^{T}	*P. jessenii* SG / *P. fluorescens* G
Pseudomonas sp. GM48	98.36	*Pseudomonas* sp. UW4	97.94		
Pseudomonas sp. GM74	97.70	*Pseudomonas* sp. GM48	97.66		
Pseudomonas sp. GM55	98.00	*Pseudomonas* sp. GM33	97.94		
Pseudomonas sp. GM33	98.72	*Pseudomonas* sp. UW4	97.97		
Pseudomonas sp. GM78	98.58	*P. umsongensis* LMG 21317^{T}	96.91		
P. fluorescens Pf0-1	96.46	*P. koreensis* LMG 21318^{T}	96.46	*P. koreensis* LMG 21318^{T}	*P. koreensis* SG / *P. fluorescens* G
Pseudomonas sp. GM30	98.33	*P. fluorescens* R124	97.78		
P. fluorescens R124	98.33	*Pseudomonas* sp. GM30	97.53		

P. fluorescens NZ011	97.11	*Pseudomonas* sp. GM80	96.80		
Pseudomonas sp. GM80	97.11	*P. fluorescens* NZ011	95.97		
Pseudomonas sp. GM50	99.51	*Pseudomonas* sp. GM102	96.83	*P. mandelii* LMG 21607[T]	*P. mandelii* SG / *P. fluorescens* G
Pseudomonas sp. GM18	97.84	*Pseudomonas* sp. GM50	96.49		
P. mandelii JR-1	99.81	*P. mandelii* LMG 21607[T]	99.81		
Pseudomonas sp. GM60	99.35	*Pseudomonas* sp. GM67	96.06		
Pseudomonas sp. GM21	97.36	*P. lini* CFBP 5737[T]	96.94		
P. fluorescens NCIMB 11764	96.96	*Pseudomonas* sp. GM21	96.38		
Pseudomonas sp. GM79	99.54	*Pseudomonas* sp. GM102	96.71		
Pseudomonas sp. GM102	99.54	*Pseudomonas* sp. GM79	96.80		
Pseudomonas sp. GM67	99.35	*Pseudomonas* sp. GM60	96.38		
Pseudomonas sp. GM17	99.05	*P. chlororaphis* subsp. *aureofaciens*30-84	99.02	*P. chlororaphis* subsp. *chlororaphis* ATCC 9446[T]	*P. chlororaphis* SG / *P. fluorescens* G
P. chlororaphis subsp. *aureofaciens* 30-84	99.05	*Pseudomonas* sp GM17	98.83		
P. protegens Pf-5	99.59	*P. protegens* DSM 19095[T]	94.15		
P. chlororaphis 06	99.73	*P. chlororaphis* subsp. *chlororaphis* PG72	98.16		
P. chlororaphis subsp. *chlororaphis* PG72	99.86	*P. chlororaphis* subsp. *aureofaciens* LMG 1245[T]	98.00		
P. fluorescens Q8r1-96	99.92	*P. brassicaceae* subsp. *brassicaceae* NFM421	95.23	*P. corrugata* ATCC 29736[T]	*P. corrugata* SG / *P. fluorescens* G
P. brassicaceae subsp. *brassicaceae* NFM421	99.92	*P. fluorescens* Q8r1-96	95.29		
P. fluorescens Q2-87	97.05	*Pseudomonas brassicaceae* subsp. *brassicaceae* NFM 421	95.03		
P. fuscovaginae CB98818	99.95	*P. fuscovaginae* LMG 25941[T]	99.27	*P. asplenii* LMG 2137[T]	*P. asplenii* SG / *P. fluorescens* G
P. fuscovaginae UPB0736	99.65	*P. fuscovaginae* LMG 25941[T] / *P. fuscovaginae* CB98818	99.46		
Pseudomonas sp. M47T1	91.01	*P. koreensis* LMG 21318[T]	91.04	*P. fluorescens* R124	n.a. SG / *P. fluorescens* G
P. savastanoi pv. *savastonoi* NCPPB 3335	99.92	*P. savastanoi* LMG 2209[T]	95.87	*P. syringae* ATCC 19310[T]	*P. syringae* G
P. syringae pv. *phaesicola* 1448A	99.02	*P. ficuserectae* CCUG 32779[T]	96.18		
P. syringae B728a	98.72	*P. syringae* ATCC 19310[T]	98.72		
P. viridiflava UASWS0038	99.97	*P. viridiflava* ATCC 13223[T]	92.87		

P. avellanae BPIC 631	99.95	*P. avellanae* CIP 105176^T	94.63		
P. syringae pv. *tomato* DC3000	98.78	*P. avellanae* BPIC 631	94.66		
P. entomophila L48^T	100.0	*P. entomophila* L48^T	92.60		
P. putida KT2440	99.35	*P. putida* BIRD-1	95.44	*P. putida* ATCC 12633^T	*P. putida* G
P. monteilii QM	99.97	*P. monteilii* ATCC 700476^T	95.61		
P. putida W619	95.33	*Pseudomonas* sp. GM84	93.64		
P. putida GB-1	97.09	*P. putida* KT2440	95.93		
P. putida S16	98.47	*P. putida* HB3267	95.10		
P. putida CSV86	94.97	*P. japonica* JCM 21532^T	90.32		
P. putida HB3267	98.47	*P. putida* S16	95.41		
P. putida BIRD-1	99.35	*P. putida* KT2440	95.13		
Pseudomonas sp. TJI-51	94.92	*P. putida* HB3267	94.11		
Pseudomonas sp. GM84	96.47	*P. plecoglossicida* ATCC 700383^T	93.58		
P. putida B6-2	99.65	*P. putida* ND6	95.41		
P. putida ND6	99.87	*P. putida* F1	95.50		
P. putida F1	99.87	*P. putida* ND6	95.41		
P. putida LS46	99.68	*P. putida* ND6	95.38		
P. fulva 12-X	96.47	*P. straminea* LMG 21615^T	96.47	*P. straminea* LMG 21615^T	*P. straminea* G
P. aeruginosa E2	99.70	*P. aeruginosa* LCT-PA102	99.19	*P. aeruginosa* ATCC10145^T	*P. aeruginosa* G
P. aeruginosa CIG1	99.95	*P. aeruginosa* ATCC 25324	99.38		
P. aeruginosa UCBPP-PA14	99.97	*P. aeruginosa* CI27	98.91		
P. aeruginosa PABLO56	99.89	*P. aeruginosa* DQ8	99.24		
P. aeruginosa N002	99.89	*P. aeruginosa* 213BR	99.38		
P. aeruginosa MPA01/P2	100.00	*P. aeruginosa* PA0579	99.43		
P. aeruginosa PA0579	100.00	*P. aeruginosa* MPA01/P2	99.43		
P. aeruginosa LCT-PA102	99.84	*P. aeruginosa* MPA01/P2 - PA0579 - ATCC 25324 - ATCC 14886 – 213BR	99.38		

P. aeruginosa LESB58	99.95	*P. aeruginosa* M18	99.40		
P. aeruginosa ATCC 25324	99.95	*P. aeruginosa* CIG1	99.43		
P. aeruginosa PA7	97.78	*P. aeruginosa* M18	97.28		
P. aeruginosa 39016	100.0	*P. aeruginosa* NCGM2.S1	99.08		
P. aeruginosa ATCC 14886	99.89	*P. aeruginosa* MPA01/P2 - PA0579 - ATCC 25324 – 213BR	99.43		
P. aeruginosa PAb1	99.84	*P. aeruginosa* PABLO56	99.35		
P. aeruginosa 138244	99.97	*P. aeruginosa* M18	99.43		
P. aeruginosa 9BR	99.97	*P. aeruginosa* 213BR	99.40		
P. aeruginosa NCMG1179	99.76	*P. aeruginosa* MPA01/P2 - PA0579 - LCT-PA102 - ATCC 25324 - ATCC 14886 – 213BR	99.30		
P. aeruginosa M18	99.97	*P. aeruginosa* 138244	99.46		
P. aeruginosa PACS2	99.78	*P. aeruginosa* LESB58 - M18	99.35		
P. aeruginosa 213BR	99.97	*P. aeruginosa* 9BR	99.43		
P. aeruginosa NCGM2.S1	100.00	*P. aeruginosa* 39016	99.08		
P. aeruginosa CI27	99.97	*P. aeruginosa* UCBPP-PA14	98.94		
P. aeruginosa DQ8	99.89	*P. aeruginosa* PABLO56	99.24		
P. aeruginosa PAO1	99.97	*P. aeruginosa* MPA01/P2 - PAO579	99.40		
P. pseudoalcaligenes KF707	91.63	*P. citronellolis* LMG 18378^T	88.67		
P. aeruginosa MRW44.1	99.97	*P. aeruginosa* MPA01/P2 - PAO579	99.40		
P. mendocina ymp	99.43	*P. mendocina* DLHK	93.80	*P. oleovorans* ATCC 8062^T	*P. oleovorans* G
P. mendocina NK-01	99.27	*P. mendocina* ATCC 25411^T	94.13		
P. mendocina DLHK	99.43	*P. mendocina* ymp	93.92		
P. pseudoalcaligenes CECT 5344	99.81	*P. oleovorans* subsp. *oleovorans* ATCC 8062^T	99.81		
P. psychrotolerans L19	99.26	*P. oleovorans* MOIL14HWK12	99.10	*P. oryzihabitans* ATCC 43272^T	*P. oryzihabitans* G
P. oleovorans MOIL14HWK12	99.54	*P. psychrotolerans* LMG 21977^T	99.40		
P. stutzeri NF13	93.28	*P. stutzeri* CCUG 29243	92.57	*P. stutzeri* ATCC 17588^T	*P. stutzeri* G
P. stutzeri SDM-LAC	94.81	*P. xanthomarina* CCUG 45643^T	88.72		

P. stutzeri DSM 10701 (JM300)	91.27	*P. stutzeri* TS44	90.45		
P. stutzeri ATCC 14445 (ZoBell)	92.27	*P. stutzeri* RCH2	90.55		
P. stutzeri RCH2	92.87	*P. stutzeri* CCUG 29243	92.54		
P. stutzeri TS44	95.18	*Pseudomonas* sp. Chol1	92.06		
P. stutzeri CCUG 29243 (AN10)	93.28	*P. stutzeri* NF13	91.34		
P. stutzeri DSM 4166 (CMT.9.A)	99.62	*P. stutzeri* T13	99.48		
P. stutzeri ATCC 17588^{T}	99.95	*P. stutzeri* ATCC 17588^{T}	99.95		
P. stutzeri A1501	99.84	*P. stutzeri* A1501	98.97		
P. stutzeri T13	99.62	*P. stutzeri* DSM 4166	99.32		
Pseudomonas sp. Chol1	95.18	*P. stutzeri* TS44	91.94		
P. stutzeri XLDN-R	99.54	*P. stutzeri* ATCC 17588^{T}	99.54		
P. agarici NCBPP2289	99.95	*P. agarici* ATCC 25941^{T}	-	-	-

n.a., not assigned.
