# Supplementary material for: Phylogenomics and systematics in Pseudomonas
Source: Front Microbiol. 2015 Mar 18;6:214. doi: 10.3389/fmicb.2015.00214 (PMC4447124; doi:10.3389/fmicb.2015.00214)
Supplement: Supplementary file 3 [file Table3.PDF]

Supplementary Table 3. Correlation coefficients between all the 6,328 pairwise whole-genome comparison results and MLSA phylogenetic similarities. In all cases correlation coefficients are significant, with significance levels of 0.

	Correlation index	ANIb	GGDC	TETRA	ANIm	MLSA
ANIb	Pearson	1	0.940	0.635	0.976	0.917
	Kendall's tau	1	0.880	0.602	0.759	0.786
	Spearman's rho	1	0.971	0.775	0.900	0.933
GGDC	Pearson	0.940	1	0.495	0.985	0.766
	Kendall's tau	0.880	1	0.601	0.769	0.788
	Spearman's rho	0.971	1	0.767	0.902	0.928
TETRA	Pearson	0.635	0.495	1	0.567	0.650
	Kendall's tau	0.602	0.601	1	0.596	0.542
	Spearman's rho	0.775	0.767	1	0.754	0.717
ANIm	Pearson	0.976	0.985	0.567	1	0.838
	Kendall's tau	0.759	0.769	0.596	1	0.721
	Spearman's rho	0.900	0.902	0.754	1	0.873
MLSA	Pearson	0.917	0.766	0.650	0.838	1
	Kendall's tau	0.786	0.788	0.542	0.721	1
	Spearman's rho	0.933	0.928	0.717	0.873	1
