# Supplementary material for: Phylogenomics and systematics in Pseudomonas
Source: Front Microbiol. 2015 Mar 18;6:214. doi: 10.3389/fmicb.2015.00214 (PMC4447124; doi:10.3389/fmicb.2015.00214)
Supplement: Supplementary file 4 [file Table4.PDF]

Supplementary Table 4. Correlation coefficients between MLSA and ANIb for the 26, 6 and 13 strains studied in the *P. aeruginosa* (325 pairwise comparisons), *P. putida* (120 pairwise comparisons) and *P. stutzeri* (91 pairwise comparisons) phylogenetic groups. In all cases correlation coefficients are significant, with significance levels of 0.

	Pearson	Kendall's tau	Spearman's rho
P. aeruginosa Group	0.976	0.599	0.770
P. putida Group	0.983	0.919	0.988
P. stutzeri Group	0.971	0.812	0.936
